# Results of silicone oil removal in post-cytomegalovirus retinitis-related retinal detachment

**DOI:** 10.1007/s12348-012-0060-3

**Published:** 2012-02-15

**Authors:** Vivek Pravin Dave, Annie Mathai, Rajeev R. Pappuru

**Affiliations:** Smt. Kanuri Santhamma Center for Vitreo Retinal Diseases, LV Prasad Eye Institute, Hyderabad, 500034 India

**Keywords:** CMV retinitis, Retinal detachment, Silicone oil removal, CD4 cell counts

## Abstract

**Purpose:**

The purpose of this study is to evaluate the results of silicone oil removal (SOR) in post-cytomegalovirus (CMV) retinitis-related retinal detachment in Indian eyes.

**Methods:**

A retrospective review of CMV retinitis-related retinal detachment following SOR was conducted.

**Results:**

Out of 60 eyes (55 patients) that had vitrectomy with silicone oil tamponade between 1995 and 2010, 11 eyes underwent SOR. There were two females and eight males, and the mean age was 41.27 ± 9.8 years. The duration between primary detachment surgery and SOR was 8.82 ± 2.56 months. The mean duration of HIV positivity was 33 ± 28.65 months (nearly 3 years). The average CD4 cell count at the time of SOR was 365 ± 85 cells/mm3. The mean best corrected pre-SOR visual acuity in logMAR was 1 ± 0.85, and the post-SOR acuity was 1.13 ± 0.67 (*p* = 0.69). The mean follow-up post-SOR was 7.54 ± 7.76 months. Out of the 11 eyes that underwent SOR, in 9 eyes, the retina was attached at the last follow-up. The anatomical success rate following SOR was 81.82% (9/11; CI, 59.03–100%).

**Conclusion:**

The results of this study suggest a relatively low redetachment rate following SOR in CMV retinitis-related retinal detachment.

## Introduction

Cytomegalovirus (CMV) retinitis is a well-recognized cause of retinal detachment in immunocompromised patients with acquired immunodeficiency syndrome (AIDS). Retinal detachments in these cases occur due to necrotic breaks at the junction of healed retinitis and normal retina. As the breaks are usually multiple, irregular, in thin retina, and difficult to visualize, primary vitrectomy with long-term endotamponade with silicone oil is the preferred modality of management in these detachments [1–4]. Though good efficacy of gas tamponade with an encircling scleral buckle has been shown in a previous study with almost 83% success rate, the total cohort was very small (six eyes). Primary vitrectomy still remains the treatment option of choice [5, 6]. Due to the inherent drawbacks of silicone oil in situ like hyperopic shift and cataractogenesis, silicone oil removal (SOR) is desirous in these cases at an optimal time [7, 8]. Though safety of silicone oil removal in post-CMV retinitis-related retinal detachment surgeries has been described before, the literature on this subset of cases is scarce [9, 10]. The purpose of this article is to assess the outcome of silicone oil removal in cases of CMV retinitis-related retinal detachment that were repaired by vitrectomy with silicone oil tamponade.

## Materials and methods

This study was a retrospective noncomparative interventional case series. The records of patients with CMV retinitis-related retinal detachment that underwent pars plana vitrectomy with silicone oil injection at a tertiary eye care institute in south India between January 1995 and October 2010 were retrospectively reviewed. The criterion used to plan oil removal was a minimum interval of 6 months following the primary vitrectomy and a completely attached retina without any unlasered retinal breaks. In all cases, silicone oil removal was planned once emulsification was noted to have begun. All patients gave a written informed consent. All surgeries were performed by or under direct supervision of two senior retinal surgeons (A.M. and R.R.P). The procedure consisted of a two-port setup, and SOR was done using a 19-G cannula on a syringe under negative suction. After SOR, the retina was examined on table with an endoilluminator to rule out any recurrent detachment and to look for adequacy of endolaser. Additional laser was applied on table as deemed necessary by making an additional sclerotomy port. Strict aseptic precautions were adhered to during surgery with adequate protective gear for the surgical and assisting team and disposal of all materials used during the surgery to prevent any risk of HIV transmission.

The data collected from the patient’s records included the patient’s age, gender, preoperative best-corrected visual acuity, postoperative best-corrected visual acuity, the type of anti-CMV treatment used, use of encirclage at the time of primary detachment surgery, time interval between the initial detachment surgery and SOR, viscosity of the silicone oil used, and whether or not SOR was combined with cataract surgery. The outcome measure studied was the number of eyes with attached retina following silicone oil removal at the last follow-up. The statistical analysis was done using the Statistical Package for Social Sciences version 16. The preoperative and postoperative visual acuity were compared using the paired *t* test. A *p* value of <0.05 was assigned as significant. The confidence intervals around the percentages were calculated by the mid-*P* exact test.

## Results

During the study period, there were 60 eyes of 55 patients who underwent primary vitrectomy with silicone oil tamponade for repair of CMV retinitis-related retinal detachment. Of these, 11 eyes of ten patients underwent SOR. Of the remaining eyes, 37 eyes were lost to follow-up before 6 months, while the remaining 12 had recurrent detachment. There were two females and eight males, and the mean age was 41.27 ± 9.8 years. The duration between primary detachment surgery and SOR was 8.82 ± 2.56 months. The mean duration of HIV positivity was 33 ± 28.65 months (nearly 3 years). The average CD4 cell count at the time of SOR was 365 ± 85 cells/mm^3^. The mean best-corrected pre-SOR visual acuity in logMAR was 1 ± 0.85, and the post-SOR acuity was 1.13 ± 0.67 (*p* = 0.69). The mean preoperative intraocular pressure (IOP) noted was 16.9 ± 2.88 mm Hg, and the postoperative IOP was 13.63 ± 2.34 mm Hg. One eye had an encirclage band passed before vitrectomy. Three eyes had active CMV retinitis when detachment developed, while the rest had healed CMV lesions. Those with active disease were put on oral Valgancyclovir 900 mg twice a day. The CD4 counts were recorded to monitor the immune status. Six out of the ten patients were on highly active antiretroviral therapy (HAART) at presentation. The rest were examined by the internist and started on HAART subsequently. Four eyes had 5,000 cs silicone oil used, while seven eyes had 1,000 cs oil.

Only one eye had cataract at the time of SOR, but cataract surgery was not performed as it was not clinically significant. Following SOR, eight eyes underwent cataract surgery over the next year. Though all eyes had an attached retina immediately following SOR, redetachment occurred in two eyes at 1 month post-SOR. One of the eyes underwent additional endolaser with silicone oil injection. The other eye had no light perception and was left alone. The causes of poor final visual acuity were multifactorial (Table 1). The mean follow-up post-SOR was 7.54 ± 7.76 months (range, 1–23 months; median, 5 months). Out of 11 eyes that underwent SOR, in 9 eyes, the retina was attached at the last follow-up. The anatomical success rate following SOR was 81.82% (9/11; CI, 59.03–100%).

**Table 1:** Causes of poor vision post-silicone oil removal

Patient number	Redetachment	Optic atrophy	Epiretinal membrane	Corneal scar	Vitritis	Macular scarring
1	−	+	−	−	−	+
2	+	+	−	−	−	−
3	+	−	−	+	−	−
4	−	−	−	−	−	−
5	−	−	−	−	−	−
6	−	−	+	−	−	−
7	−	−	−	−	+	+
8	−	+	+	−	+	+
9	−	−	+	+	−	+
10	−	+	−	−	−	−
11	−	+	−	−	−	−

## Discussion

Silicone oil is the preferred tamponade following vitrectomy for retinal detachment surgeries associated with CMV retinitis. A wide range of retinal attachment rate varying from 8–95% has been reported in the literature for these cases with silicone oil tamponade [1, 9] With the advent of the HAART therapy for HIV patients, the life expectancy has increased over a period of time. Thus, an increased longevity necessitates SOR in many patients who have undergone primary vitreous surgery with silicone oil tamponade. As there is paucity of literature on the outcomes of such eyes following SOR, our study aimed to document the outcomes of these eyes. A similar study conducted by Morrison et al. [10] reported a 53% rate of redetachment after silicone oil removal at a median period of 4 months post-SOR. Nine eyes in their series had C3F8, SF6, or air injection at the end of surgery. They observed that simultaneous cataract surgery at the time of SOR and lower CD4 cell counts were associated with a greater risk of redetachment. Our study showed a redetachment rate of 18.2% (2/11; 95% CI, 0–41%). The mean CD4 cell count in their study was 173 cells/mm^3^, while that in our study was 365 cells/mm^3^. Also, none of our patients had cataract surgery combined with the SOR at the same sitting. The high CD4 cell count indicates a greater immune competence and possibly could be hypothesized to have contributed to better surgical outcome [11].

Our study has a few inherent limitations and observations. Being a retrospective study, the results need to be interpreted with caution. As the overall sample size is small (11 eyes) and the number of unfavorable events being still smaller (two redetachments), to derive any statistical inference from this study would be erroneous. The overall follow-up is short, both after the primary surgery and after silicone oil removal. This is possibly due to the majority of patients belonging to the lower socioeconomic strata as well as the high mortality associated with AIDS. Among the eyes that did not undergo SOR, 12 eyes had recurrent detachment. A high rate of loss of follow-up (62%) is a major limiting factor of the present study.

In summary, among the eyes that did not undergo SOR, 12 eyes had recurrent detachment. Overall, 14 out of 23 eyes (61%; CI, 40–79%) developed recurrent retinal detachment on follow-up. Our series also showed a retinal attachment rate of 82% following silicone oil removal at a mean follow-up of 7.5 months in eyes that underwent vitrectomy for CMV retinitis-related retinal detachment. This is an improvement on the rates that have been reported in the literature and could be attributed to the higher CD4 counts. Thus, though the overall redetachment rate post-vitrectomy with silicone oil is poor, in cases with attached retina over 6 months, there is a high attachment rate seen post-SOR. A larger prospective series with a longer follow-up is desirous to further validate these results.
